# Crystal structure of *N*-(di­phenyl­phosphor­yl)-2-meth­oxy­benzamide

**DOI:** 10.1107/S205698901900762X

**Published:** 2019-06-04

**Authors:** Victor A. Trush, Nataliia S. Kariaka, Viktoriya V. Dyakonenko, Svitlana V. Shishkina, Vladimir M. Amirkhanov

**Affiliations:** a Department of Chemistry, Kyiv National Taras Shevchenko University, Volodymyrska, str. 64, 01601 Kyiv, Ukraine; b SSI "Institute for Single Crystals", National Academy of Sciences of Ukraine, Nauki Ave 60, Kharkiv 61001, Ukraine

**Keywords:** crystal structure, carbacyl­amido­phosphate

## Abstract

In the title compound, C_20_H_18_NO_3_P, the C=O and P=O groups of the carbacyl­amido­phosphate (CAPh) fragments are located in a synclinal position relative to each other and are pre-organized for bidentate chelate coordination of metal ions. The N—H group is involved in the formation of an intra­molecular hydrogen bond. In the crystal, mol­ecules do not form strong inter­molecular inter­actions but the mol­ecules are linked *via* weak C—H⋯π inter­actions, forming chains along [001].

## Abstract 2

In the title compound, C_20_H_18_NO_3_P, the C=O and P=O groups of the carbacyl­amido­phosphate (CAPh) fragments are located in a synclinal position relative to each othe. The N—H group is involved in the formation of an intra­molecular hydrogen bond.

## Chemical context   

P,N-substituted analogues of β-diketones, carbac­ylamido­phosphates (CAPh) (Amirkhanov *et al.*, 2014 ▸) that contain a C(O)NHP(O) structural fragment are known for their wide range of biological activity (Adams *et al.*, 2002 ▸; Grimes *et al.*, 2008 ▸; Grynyuk *et al.*, 2016 ▸). They act as powerful chelating ligands (Skopenko *et al.*, 2004 ▸; Amirkhanov *et al.*, 2014 ▸) and as lanthanide luminescence sensitizers (Kariaka *et al.*, 2016 ▸; Pham *et al.*, 2017 ▸; Kariaka *et al.*, 2018 ▸). Thus, the syntheses and structure analysis of CAPhs are of increased inter­est and some structural and conformation studies of related CAPh type mol­ecules were reported recently (Breuer *et al.*, 1990 ▸; Amirkhanov *et al.*, 1997 ▸; Milton *et al.*, 2004**a* ▸,*b* ▸;* Kariaka *et al.*, 2014 ▸). Herein we report synthesis and crystal structure of a new CAPh, *N*-(di­phenyl­phosphor­yl)-2-meth­oxy­benzamide (I).
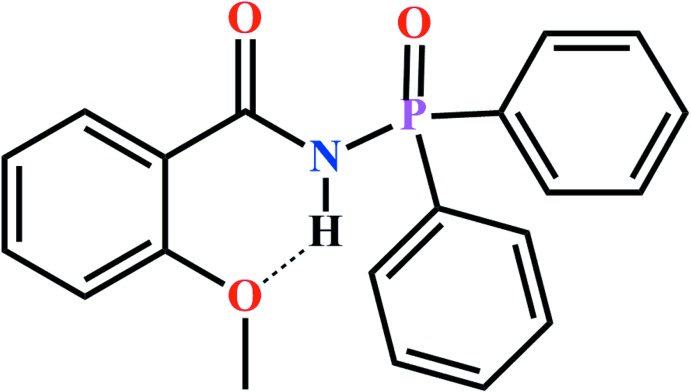


## Structural commentary   

**Figure 1:**
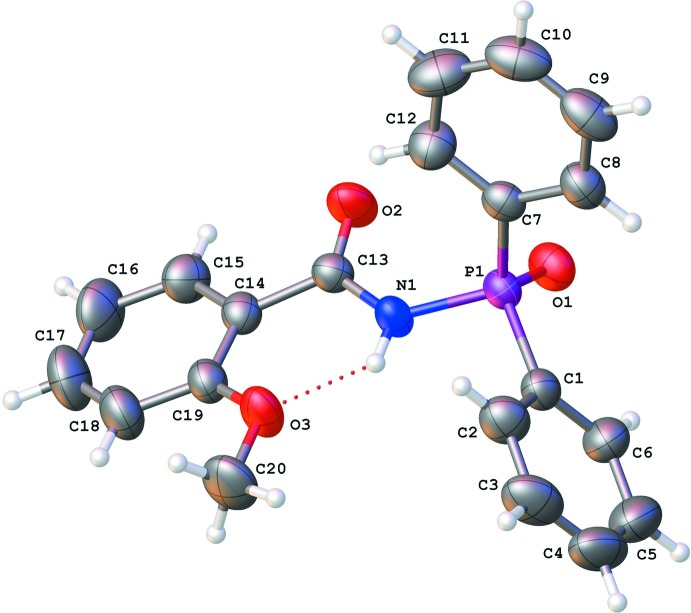
The mol­ecular structure of the title compound with displacement ellipsoids drawn at the 50% probability level.

The mol­ecular structure of the title compound is shown in Fig. 1 ▸. The bonds lengths in the C(O)NHP(O) fragment are typical for CAPh type ligands. The C=O and P=O groups are located in a synclinal position relatively to each other as evidenced by the O1—P1—N1—C13 torsion angle of −45.5 (2)°, O2—C13—N1—P1 torsion angle of −2.7 (3)°, and the O1—P1⋯C13—O2 pseudo-torsion angle of −42.9 (2)°. As a result the CAPh fragment conformation is pre-organized for bidentate chelate coordination of metal ions.

The conjugation between the carbamide group and the anisole substituent is broken, as evidenced by the value of the C13—C14 bond length of 1.496 (3) Å, which is comparable to the mean value for non-conjugated C_ar_—C*sp* ^2^ bonds (1.488 Å; Burgi & Dunitz, 1994 ▸). At the same time, the anisole and carbamide fragments are slightly non-coplanar. The C19—C14—C13—N1 torsion angle is 13.6 (3)° despite the formation of the N1—H1⋯O3 strong enough hydrogen bond (the H1⋯O3 distance is 1.93 Å and the N1—H1⋯O3 angle is 137°; Table 1 ▸). The methyl group of the meth­oxy substituent lies in the plane of the attached benzene ring despite the significant steric repulsion [the shortened intra­molecular contacts are: H20*A*⋯H18 = 2.26 Å (the sum of the vdW radii is 2.32 Å; Zefirov, 1997 ▸), H20*C*⋯H18 = 2.28 Å and C20⋯H18 = 2.48 Å (the sum of the vdW radii is 2.87 Å)]. The phospho­rus atom environment has a distorted tetra­hedral configuration. The C1–C6 phenyl ring is almost coplanar with the P=O bond [the C6—C1—P1—O1 torsion angle is −5.7 (2)°] while the C7–C12 phenyl ring is rotated significantly relatively to the P=O bond as defined by the C8—C7—P1—O1 torsion angle of −72.7 (2)°.

## Supra­molecular features   

**Figure 2:**
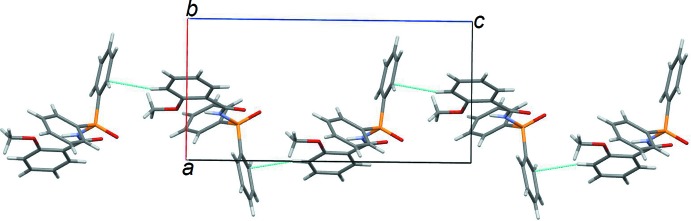
A section of a chain along the *c* axis formed by weak C—H⋯π inter­actions (shown as blue dotted lines).

It has been shown that CAPhs display different solid-state motifs (Breuer *et al.*, 1990 ▸; Amirkhanov *et al.*,1997 ▸; Milton *et al.*, 2004*a* ▸,*b* ▸; Kariaka *et al.*, 2014 ▸), *i.e*. chains, dimers and more seldom monomers. These motifs are realized through existence of hydrogen bonds with participation of the –N—H group. In crystal of (I), the –N—-H group participates in an intra­molecular hydrogen bond. There are no strong proton donors capable of forming inter­molecular hydrogen bonds in this mol­ecule. Thus the title mol­ecules form only weak C—H⋯π inter­actions leading to chains of mol­ecules along the *c*-axis direction (Figs. 2 ▸ and 3 ▸).

## Database survey   

A search of the Cambridge Structural Database (CSD, Version 5.40, update of November 2018; Groom *et al.*, 2016 ▸) for complexes containing CAPh ligands yielded 189 hits. In the CAPh fragments, the mean C=O bond length is 1.222 Å, the mean C—N bond length is 1.364 Å, the mean N—P bond length is 1.686 Å and the mean P=O bond length is 1.504 Å.

## Synthesis and crystallization   

**Figure 4:**
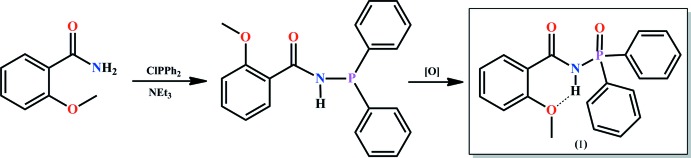
The two-step reaction for the preparation of the title compound (I).

N-(di­phenyl­phosphor­yl)-2-meth­oxy­benzamide (I) was prepared according to a two-step reaction (Fig. 4 ▸).

To a solution of *o*-meth­oxy­benzamide (1.51 g, 0.01 mol) and tri­ethyl­amine (2.03 g, 2.8 ml, 0.02 mol) in 20 ml of dioxane was added chloro­diphenyl­phosphine (2.2 g, 1.79 ml, 0.01 mol) under an inert atmosphere. The mixture was stirred under reflux for 60 min and evaporated to dryness to give a pasty residue, which was mixed with 20 ml of acetone and then a solution of 0.01 mol of H_2_O_2_ in 5 ml of acetone was added dropwise under vigorous stirring at 273 K. The brownish solution was evaporated and the residue was mixed with 50 ml of 10% aqueous HOAc. The solid precipitate was filtered, washed with cold water (2 × 20 ml) and recrystallized from *i*-PrOH [2.8 g (80%)]. Single crystals suitable for X-ray diffraction were grown from dilute *i*-PrOH solution by slow evaporation after one week.

M.p. 431–434 K. IR (KBr pellet, cm^−1^): 3271*m* [ν(NH)], 3059*w*, 3011*w*, 2985*w*, 2949*w*, 2924*w*, 2843*w*, 1671*vs* [ν(CO)], 1601*m*, 1486*w*, 1461*vs* (Amide-II), 1436*s*, 1294*m*, 1242*m*, 1225*vs* [ν(PO)], 1181*m*, 1160*w*, 1125*s*, 1109*m*, 1045*w*, 1012*m*, 868*w* [(PN)], 840*m*, 786*m*, 767*m*, 754*s*, 726*m*, 702*m*, 668*w*, 634*w*, 592*w*, 543*m*, 524*s*, 512*m*, 487*m*, 442*w*. ^1^H NMR (DMSO-*d*6): C—H 3.95 (*s*, 3H), 7.07 (*t*, 1H), 7.22 (*d*, 1H), 7.58 (*t*, 7H), 7.67 (*d*, 1H), 7.88 (*m*, 4H), N—H 9.90 (*d*, 1H) ppm. UV–Vis abs. (CH_2_Cl_2_, λ_max_, nm): 240, 295.

## Refinement   

Crystal data, data collection and structure refinement details are summarized in Table 2 ▸. H atoms were placed in calculated positions (N—H = 0.86, C—H = 0.93–0.96 Å) and refined as riding with *U*_iso_(H) = 1.2*U*_eq_(C,N) or 1.5*U*_eq_(C-meth­yl).

## Supplementary Material

Crystal structure: contains datablock(s) I. DOI: 10.1107/S205698901900762X/lh5904sup1.cif

Structure factors: contains datablock(s) I. DOI: 10.1107/S205698901900762X/lh5904Isup2.hkl

Supporting information file. DOI: 10.1107/S205698901900762X/lh5904Isup3.cml

CCDC reference: 1918613

Additional supporting information:  crystallographic information; 3D view; checkCIF report

## Figures and Tables

**Figure 3:**
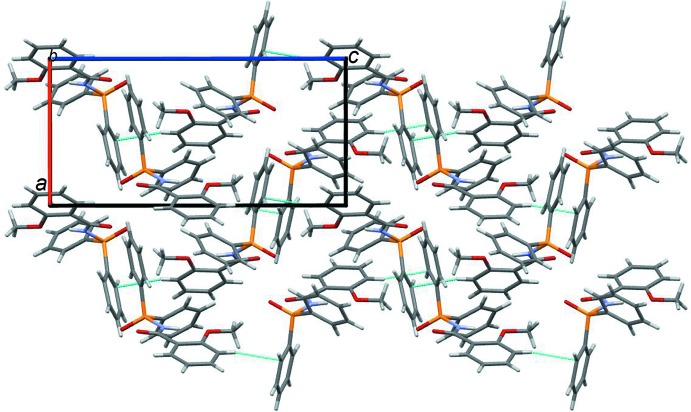
The crystal packing of the title compound viewed along the *b* axis.

**Table 1:** Hydrogen-bond geometry (Å, °)

*D*—H⋯*A*	*D*—H	H⋯*A*	*D*⋯*A*	*D*—H⋯*A*
N1—H1⋯O3	0.86	1.93	2.628 (2)	137
C18—H18⋯*Cg* ^i^	0.93	2.99	3.864 (3)	158

Symmetry code: (i) 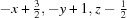.

**Table 2:** Experimental details

Crystal data	
Chemical formula	C_20_H_18_NO_3_P
*M*_r_	351.32
Crystal system, space group	Orthorhombic, *P*2_1_2_1_2_1_
Temperature (K)	294
*a*, *b*, *c* (Å)	8.317 (2), 12.657 (2), 16.763 (3)
*V* (Å^3^)	1764.6 (6)
*Z*	4
Radiation type	Mo *K*α
μ (mm^−1^)	0.17
Crystal size (mm)	0.5 × 0.4 × 0.3

Data collection	
Diffractometer	Rigaku Oxford Diffraction Xcalibur, Sapphire3
Absorption correction	Multi-scan (*CrysAlis PRO*; Rigaku OD, 2018 ▸)
*T*_min_, *T*_max_	0.986, 1.000
No. of measured, independent and observed [*I* > 2σ(*I*)] reflections	18281, 5691, 4247
*R*_int_	0.032
(sin θ/λ)_max_ (Å^−1^)	0.749

Refinement	
*R*[*F* ^2^ > 2σ(*F* ^2^)], *wR*(*F* ^2^), *S*	0.043, 0.113, 1.11
No. of reflections	5691
No. of parameters	227
H-atom treatment	H-atom parameters constrained
Δρ_max_, Δρ_min_ (e Å^−3^)	0.23, −0.32
Absolute structure	Flack *x* determined using 1430 quotients [(*I* ^+^)−(*I* ^−^)]/[(*I* ^+^)+(*I* ^−^)] (Parsons et al., 2013 ▸)
Absolute structure parameter	0.01 (4)

Computer programs: *CrysAlis PRO* (Rigaku OD, 2018 ▸), *SHELXT* (Sheldrick, 2015*a* ▸), *SHELXL* (Sheldrick, 2015*b* ▸) and *OLEX2* (Dolomanov *et al.*, 2009 ▸).

## supplementary crystallographic information

### Crystal data

**Table:**

C_20_H_18_NO_3_P	*D*_x_ = 1.322 Mg m^−^^3^
*M**_r_* = 351.32	Mo *K*α radiation, λ = 0.71073 Å
Orthorhombic, *P*2_1_2_1_2_1_	Cell parameters from 5505 reflections
*a* = 8.317 (2) Å	θ = 3.2–30.6°
*b* = 12.657 (2) Å	µ = 0.17 mm^−^^1^
*c* = 16.763 (3) Å	*T* = 294 K
*V* = 1764.6 (6) Å^3^	Block, colourless
*Z* = 4	0.5 × 0.4 × 0.3 mm
*F*(000) = 736	

### Data collection

**Table:**

Rigaku Oxford Diffraction Xcalibur, Sapphire3 diffractometer	5691 independent reflections
Radiation source: fine-focus sealed X-ray tube, Enhance (Mo) X-ray Source	4247 reflections with *I* > 2σ(*I*)
Graphite monochromator	*R*_int_ = 0.032
Detector resolution: 16.1827 pixels mm^-1^	θ_max_ = 32.2°, θ_min_ = 3.2°
ω scans	*h* = −12→12
Absorption correction: multi-scan (CrysAlis PRO; Rigaku OD, 2018)	*k* = −17→16
*T*_min_ = 0.986, *T*_max_ = 1.000	*l* = −23→24
18281 measured reflections	

### Refinement

**Table:**

Refinement on *F*^2^	Hydrogen site location: inferred from neighbouring sites
Least-squares matrix: full	H-atom parameters constrained
*R*[*F*^2^ > 2σ(*F*^2^)] = 0.043	*w* = 1/[σ^2^(*F*_o_^2^) + (0.0551*P*)^2^] where *P* = (*F*_o_^2^ + 2*F*_c_^2^)/3
*wR*(*F*^2^) = 0.113	(Δ/σ)_max_ = 0.001
*S* = 1.11	Δρ_max_ = 0.23 e Å^−^^3^
5691 reflections	Δρ_min_ = −0.32 e Å^−^^3^
227 parameters	Absolute structure: Flack *x* determined using 1430 quotients [(*I*^+^)-(*I*^-^)]/[(*I*^+^)+(*I*^-^)] (Parsons et al., 2013)
0 restraints	Absolute structure parameter: 0.01 (4)

### Special details

**Table:**

Geometry. All esds (except the esd in the dihedral angle between two l.s. planes) are estimated using the full covariance matrix. The cell esds are taken into account individually in the estimation of esds in distances, angles and torsion angles; correlations between esds in cell parameters are only used when they are defined by crystal symmetry. An approximate (isotropic) treatment of cell esds is used for estimating esds involving l.s. planes.

### Fractional atomic coordinates and isotropic or equivalent isotropic displacement parameters (Å^2^)

**Table:**

	*x*	*y*	*z*	*U*_iso_*/*U*_eq_	
P1	0.75683 (6)	0.34774 (4)	0.68054 (3)	0.03788 (14)	
O1	0.8432 (2)	0.33778 (15)	0.75665 (10)	0.0522 (4)	
O2	0.8579 (2)	0.57607 (14)	0.70208 (11)	0.0601 (5)	
O3	0.8588 (2)	0.48946 (15)	0.46564 (10)	0.0574 (5)	
N1	0.8291 (2)	0.43972 (14)	0.61711 (11)	0.0420 (4)	
H1	0.839131	0.420776	0.568098	0.050*	
C1	0.7720 (3)	0.23277 (18)	0.61801 (14)	0.0454 (5)	
C2	0.6930 (4)	0.2254 (2)	0.54479 (17)	0.0585 (7)	
H2	0.624578	0.279355	0.528470	0.070*	
C3	0.7160 (4)	0.1383 (3)	0.4964 (2)	0.0768 (10)	
H3	0.665554	0.134563	0.447009	0.092*	
C4	0.8138 (5)	0.0571 (3)	0.5216 (2)	0.0815 (11)	
H4	0.827802	−0.001937	0.489292	0.098*	
C5	0.8912 (5)	0.0626 (3)	0.5942 (2)	0.0802 (10)	
H5	0.956231	0.007064	0.610905	0.096*	
C6	0.8722 (3)	0.1509 (2)	0.64224 (18)	0.0591 (6)	
H6	0.926469	0.155311	0.690643	0.071*	
C7	0.5462 (3)	0.37685 (18)	0.69283 (13)	0.0410 (5)	
C8	0.4455 (3)	0.2966 (2)	0.72018 (16)	0.0528 (6)	
H8	0.486311	0.229064	0.728194	0.063*	
C9	0.2844 (3)	0.3171 (3)	0.73552 (18)	0.0653 (8)	
H9	0.217061	0.262777	0.752171	0.078*	
C10	0.2247 (3)	0.4165 (3)	0.72626 (17)	0.0669 (8)	
H10	0.117415	0.430192	0.738074	0.080*	
C11	0.3223 (3)	0.4969 (3)	0.6995 (2)	0.0706 (9)	
H11	0.280930	0.564645	0.692976	0.085*	
C12	0.4832 (3)	0.4765 (2)	0.68216 (19)	0.0553 (6)	
H12	0.548597	0.530641	0.663257	0.066*	
C13	0.8734 (3)	0.54108 (18)	0.63499 (14)	0.0405 (5)	
C14	0.9427 (3)	0.60615 (19)	0.56888 (14)	0.0417 (5)	
C15	1.0164 (3)	0.7003 (2)	0.59123 (18)	0.0557 (6)	
H15	1.020894	0.718620	0.644915	0.067*	
C16	1.0828 (4)	0.7669 (3)	0.5352 (3)	0.0769 (10)	
H16	1.130614	0.830077	0.550753	0.092*	
C17	1.0775 (4)	0.7386 (3)	0.4553 (2)	0.0814 (10)	
H17	1.124742	0.782560	0.417536	0.098*	
C18	1.0042 (4)	0.6472 (3)	0.43069 (19)	0.0662 (7)	
H18	0.999839	0.630235	0.376750	0.079*	
C19	0.9367 (3)	0.58040 (19)	0.48673 (15)	0.0460 (5)	
C20	0.8551 (5)	0.4599 (3)	0.38290 (17)	0.0768 (9)	
H20A	0.800672	0.513521	0.352765	0.115*	
H20B	0.799139	0.393959	0.377171	0.115*	
H20C	0.963101	0.452306	0.363487	0.115*	

### Atomic displacement parameters (Å^2^)

**Table:**

	*U*^11^	*U*^22^	*U*^33^	*U*^12^	*U*^13^	*U*^23^
P1	0.0406 (3)	0.0420 (3)	0.0310 (2)	−0.0018 (2)	0.0008 (2)	0.0024 (2)
O1	0.0519 (8)	0.0658 (11)	0.0389 (8)	0.0029 (8)	−0.0059 (7)	0.0059 (8)
O2	0.0794 (12)	0.0568 (11)	0.0442 (10)	−0.0133 (9)	0.0112 (9)	−0.0149 (8)
O3	0.0811 (12)	0.0574 (11)	0.0335 (9)	−0.0146 (9)	0.0021 (9)	0.0008 (8)
N1	0.0548 (11)	0.0402 (9)	0.0311 (9)	−0.0092 (8)	0.0059 (8)	−0.0012 (7)
C1	0.0509 (12)	0.0405 (11)	0.0446 (12)	−0.0045 (9)	0.0086 (10)	0.0009 (9)
C2	0.0654 (16)	0.0594 (16)	0.0508 (15)	−0.0080 (12)	0.0012 (12)	−0.0084 (12)
C3	0.089 (2)	0.083 (2)	0.0590 (18)	−0.0218 (19)	0.0111 (16)	−0.0255 (16)
C4	0.097 (3)	0.0608 (19)	0.086 (3)	−0.0094 (17)	0.035 (2)	−0.0242 (18)
C5	0.092 (2)	0.0549 (17)	0.094 (3)	0.0149 (16)	0.027 (2)	−0.0013 (17)
C6	0.0638 (15)	0.0519 (14)	0.0616 (16)	0.0086 (13)	0.0087 (13)	0.0025 (13)
C7	0.0408 (10)	0.0489 (12)	0.0331 (11)	−0.0032 (8)	−0.0014 (8)	−0.0044 (9)
C8	0.0548 (13)	0.0565 (15)	0.0469 (14)	−0.0089 (12)	0.0052 (11)	0.0011 (11)
C9	0.0491 (14)	0.095 (2)	0.0517 (15)	−0.0219 (14)	0.0053 (11)	−0.0048 (14)
C10	0.0392 (13)	0.104 (2)	0.0573 (17)	0.0016 (15)	−0.0015 (11)	−0.0209 (16)
C11	0.0511 (15)	0.076 (2)	0.085 (2)	0.0148 (14)	−0.0076 (15)	−0.0170 (17)
C12	0.0454 (12)	0.0560 (14)	0.0646 (16)	−0.0004 (10)	−0.0037 (12)	0.0033 (13)
C13	0.0398 (10)	0.0424 (12)	0.0391 (11)	−0.0023 (9)	0.0021 (9)	−0.0032 (9)
C14	0.0362 (10)	0.0422 (11)	0.0466 (13)	−0.0018 (9)	0.0011 (9)	0.0036 (10)
C15	0.0512 (13)	0.0511 (14)	0.0648 (17)	−0.0109 (11)	0.0025 (12)	−0.0049 (12)
C16	0.0706 (19)	0.0583 (18)	0.102 (3)	−0.0270 (15)	0.0064 (18)	0.0094 (17)
C17	0.084 (2)	0.078 (2)	0.083 (3)	−0.0257 (18)	0.0138 (19)	0.0254 (19)
C18	0.0763 (18)	0.0690 (18)	0.0532 (15)	−0.0094 (16)	0.0102 (14)	0.0132 (14)
C19	0.0463 (12)	0.0458 (13)	0.0460 (13)	−0.0034 (10)	0.0053 (10)	0.0063 (10)
C20	0.107 (3)	0.086 (2)	0.0374 (14)	−0.012 (2)	0.0033 (16)	−0.0083 (14)

### Geometric parameters (Å, º)

**Table:**

P1—O1	1.4695 (17)	C8—C9	1.389 (4)
P1—N1	1.6871 (19)	C9—H9	0.9300
P1—C1	1.798 (2)	C9—C10	1.361 (5)
P1—C7	1.802 (2)	C10—H10	0.9300
O2—C13	1.215 (3)	C10—C11	1.377 (5)
O3—C19	1.368 (3)	C11—H11	0.9300
O3—C20	1.437 (3)	C11—C12	1.394 (4)
N1—H1	0.8600	C12—H12	0.9300
N1—C13	1.368 (3)	C13—C14	1.496 (3)
C1—C2	1.396 (4)	C14—C15	1.392 (3)
C1—C6	1.391 (4)	C14—C19	1.416 (3)
C2—H2	0.9300	C15—H15	0.9300
C2—C3	1.382 (4)	C15—C16	1.377 (4)
C3—H3	0.9300	C16—H16	0.9300
C3—C4	1.377 (5)	C16—C17	1.388 (5)
C4—H4	0.9300	C17—H17	0.9300
C4—C5	1.378 (6)	C17—C18	1.371 (5)
C5—H5	0.9300	C18—H18	0.9300
C5—C6	1.387 (4)	C18—C19	1.382 (4)
C6—H6	0.9300	C20—H20A	0.9600
C7—C8	1.394 (3)	C20—H20B	0.9600
C7—C12	1.378 (3)	C20—H20C	0.9600
C8—H8	0.9300		
			
O1—P1—N1	115.61 (10)	C9—C10—H10	119.8
O1—P1—C1	113.72 (11)	C9—C10—C11	120.3 (2)
O1—P1—C7	113.17 (10)	C11—C10—H10	119.8
N1—P1—C1	99.56 (10)	C10—C11—H11	120.1
N1—P1—C7	106.09 (11)	C10—C11—C12	119.8 (3)
C1—P1—C7	107.49 (11)	C12—C11—H11	120.1
C19—O3—C20	118.6 (2)	C7—C12—C11	120.5 (3)
P1—N1—H1	116.4	C7—C12—H12	119.7
C13—N1—P1	127.23 (16)	C11—C12—H12	119.7
C13—N1—H1	116.4	O2—C13—N1	121.0 (2)
C2—C1—P1	122.3 (2)	O2—C13—C14	121.7 (2)
C6—C1—P1	118.4 (2)	N1—C13—C14	117.24 (19)
C6—C1—C2	119.3 (2)	C15—C14—C13	116.2 (2)
C1—C2—H2	119.9	C15—C14—C19	118.3 (2)
C3—C2—C1	120.3 (3)	C19—C14—C13	125.4 (2)
C3—C2—H2	119.9	C14—C15—H15	119.4
C2—C3—H3	120.1	C16—C15—C14	121.1 (3)
C4—C3—C2	119.9 (3)	C16—C15—H15	119.4
C4—C3—H3	120.1	C15—C16—H16	120.4
C3—C4—H4	119.7	C15—C16—C17	119.2 (3)
C3—C4—C5	120.6 (3)	C17—C16—H16	120.4
C5—C4—H4	119.7	C16—C17—H17	119.2
C4—C5—H5	120.0	C18—C17—C16	121.5 (3)
C4—C5—C6	120.0 (3)	C18—C17—H17	119.2
C6—C5—H5	120.0	C17—C18—H18	120.3
C1—C6—H6	120.0	C17—C18—C19	119.5 (3)
C5—C6—C1	119.9 (3)	C19—C18—H18	120.3
C5—C6—H6	120.0	O3—C19—C14	117.5 (2)
C8—C7—P1	118.24 (19)	O3—C19—C18	122.1 (2)
C12—C7—P1	122.82 (18)	C18—C19—C14	120.4 (2)
C12—C7—C8	118.8 (2)	O3—C20—H20A	109.5
C7—C8—H8	119.8	O3—C20—H20B	109.5
C9—C8—C7	120.3 (3)	O3—C20—H20C	109.5
C9—C8—H8	119.8	H20A—C20—H20B	109.5
C8—C9—H9	119.9	H20A—C20—H20C	109.5
C10—C9—C8	120.2 (3)	H20B—C20—H20C	109.5
C10—C9—H9	119.9		
			
P1—N1—C13—O2	−2.7 (3)	C3—C4—C5—C6	−0.6 (5)
P1—N1—C13—C14	176.74 (16)	C4—C5—C6—C1	1.5 (5)
P1—C1—C2—C3	176.0 (2)	C6—C1—C2—C3	−0.9 (4)
P1—C1—C6—C5	−177.8 (2)	C7—P1—N1—C13	80.8 (2)
P1—C7—C8—C9	175.8 (2)	C7—P1—C1—C2	51.3 (2)
P1—C7—C12—C11	−174.1 (2)	C7—P1—C1—C6	−131.8 (2)
O1—P1—N1—C13	−45.5 (2)	C7—C8—C9—C10	−2.0 (4)
O1—P1—C1—C2	177.4 (2)	C8—C7—C12—C11	0.9 (4)
O1—P1—C1—C6	−5.7 (2)	C8—C9—C10—C11	1.8 (4)
O1—P1—C7—C8	−72.7 (2)	C9—C10—C11—C12	−0.3 (5)
O1—P1—C7—C12	102.2 (2)	C10—C11—C12—C7	−1.0 (5)
O2—C13—C14—C15	12.0 (3)	C12—C7—C8—C9	0.6 (4)
O2—C13—C14—C19	−166.9 (2)	C13—C14—C15—C16	−179.3 (2)
N1—P1—C1—C2	−59.0 (2)	C13—C14—C19—O3	1.2 (4)
N1—P1—C1—C6	117.90 (19)	C13—C14—C19—C18	179.5 (2)
N1—P1—C7—C8	159.47 (19)	C14—C15—C16—C17	−0.8 (5)
N1—P1—C7—C12	−25.6 (2)	C15—C14—C19—O3	−177.7 (2)
N1—C13—C14—C15	−167.5 (2)	C15—C14—C19—C18	0.6 (4)
N1—C13—C14—C19	13.6 (3)	C15—C16—C17—C18	1.7 (6)
C1—P1—N1—C13	−167.8 (2)	C16—C17—C18—C19	−1.4 (5)
C1—P1—C7—C8	53.7 (2)	C17—C18—C19—O3	178.4 (3)
C1—P1—C7—C12	−131.3 (2)	C17—C18—C19—C14	0.2 (4)
C1—C2—C3—C4	1.8 (5)	C19—C14—C15—C16	−0.3 (4)
C2—C1—C6—C5	−0.7 (4)	C20—O3—C19—C14	−178.1 (3)
C2—C3—C4—C5	−1.0 (5)	C20—O3—C19—C18	3.6 (4)

### Hydrogen-bond geometry (Å, º)

**Table:**

*D*—H···*A*	*D*—H	H···*A*	*D*···*A*	*D*—H···*A*
N1—H1···O3	0.86	1.93	2.628 (2)	137
C18—H18···*Cg*^i^	0.93	2.99	3.864 (3)	158

Symmetry code: (i) −*x*+3/2, −*y*+1, *z*−1/2.
